# Plasma Exosomes of Patients with Breast and Ovarian Tumors Contain an Inactive 20S Proteasome

**DOI:** 10.3390/molecules26226965

**Published:** 2021-11-18

**Authors:** Natalia Yunusova, Elena Kolegova, Elena Sereda, Larisa Kolomiets, Alisa Villert, Marina Patysheva, Irina Rekeda, Alina Grigor’eva, Natalia Tarabanovskaya, Irina Kondakova, Svetlana Tamkovich

**Affiliations:** 1 Tomsk National Research Medical Center, Cancer Research Institute, Russian Academy of Sciences, 634009 Tomsk, Russia; Bochkarevanv@oncology.tomsk.ru (N.Y.); elenakolegova@oncology.tomsk.ru (E.K.); SchaschovaEE@oncology.tomsk.ru (E.S.); KolomietsLA@oncology.tomsk.ru (L.K.); Avillert@yandex.ru (A.V.); starin5@yandex.ru (M.P.); tarabanovskaya@inbox.ru (N.T.); kondakova@oncology.tomsk.ru (I.K.); 2 Department of Biochemistry and Molecular Biology, Faculty of Medicine and Biology, Siberian State Medical University, 634050 Tomsk, Russia; 3 Laboratory for Translational Cell and Molecular Biomedicine, National Tomsk State University, 634050 Tomsk, Russia; 4 Department of Mammology, Novosibirsk Regional Clinical Oncological Dispensary, 630108 Novosibirsk, Russia; rekeda84@mail.ru; 5 Institute of Chemical Biology and Fundamental Medicine, Siberian Branch of Russian Academy of Sciences, 630090 Novosibirsk, Russia; feabelit@mail.ru; 6 Department of Clinical Biochemistry, V. Zelman Institute for Medicine and Psychology, Novosibirsk State University, 630090 Novosibirsk, Russia

**Keywords:** exosomes, 20S proteasome, plasma, tissue, breast cancer, ovarian cancer

## Abstract

Exosomes are directly involved in governing of physiological and pathological conditions of an organism through the transfer of information from producing to receiving cells. It can be assumed that exosomes are one of the key players of tumor dissemination since they are very stable and small enough to penetrate from various tissues into biological fluids and then back, thus interacting with tissue target cells. We evaluated the enzymatic activity and the level of 20S proteasome in tissue and exosomes of healthy females (*n* = 39) and patients with ovarian (*n* = 50) and breast (*n* = 108) tumors to reveal the critical role of exosomal cargo in the mediation of different types of metastases. Exosomes from plasma and ascites were isolated and characterized in according to International Society for Extracellular Vesicles guidelines. The level of 20S proteasome in tissue and exosomes was determined using Western blot analysis. Chymotrypsin- and caspase-like (ChTL and CL, respectively) peptidase activities of the proteasomes were determined using fluorogenic Suc-LLVY-AMC and Cbz-LLG-AMC substrates, respectively. We observed increased levels of 20S proteasome in ovarian cancer tissue and luminal B subtype breast cancer tissue as well as in plasma exosomes from cancer patients. Moreover, the level of the 20S proteasome in plasma exosomes and ascites exosomes in patients with ovarian tumors is comparable and higher in ovarian cancer patients with low volume ascites than in patients with moderate and high-volume ascites. We also found increased ChTL and CL activities in breast cancer and ovarian cancer tissues, as well as in peritoneal metastases in ovarian cancer, while proteasomal activity in exosomes from plasma of healthy females and all patients, as well as from ascites of ovarian tumor patients were lower than detection limit of assay. Thus, regardless of the type of tumor metastasis (lymphogenous or peritoneal), the exosomes of cancer patients were characterized by an increased level of 20S proteasome, which do not exhibit enzymatic activity.

## 1. Introduction

The study of the mechanisms of the metastases generation at the molecular level is necessary for the development of diagnostic approaches for predicting the risk of metastasis, prevention, and treatment of metastatic disease. Currently, it has been proven that one of the leading roles in metastasis belongs to proteases. These enzymes degrade the extracellular matrix, disrupt adhesive contacts, and induce angiogenesis [1]. It is known that the ubiquitin–proteasome system is the most important system of specific intracellular proteolysis, providing degradation of proteins in a wide range of cellular pathways [2]. In tissues, proteolysis occurs directly in the 20S proteasome, the core of which consists of four folded heptamer rings, including two outer α-rings and two inner β-rings. Activation of the 20S proteasome occurs through the attachment of regulatory particles to external α-subunits (19S, PA28). The two outer rings are composed of homologous α-subunits (α1α2α3α4α5α6α7). The N-terminal domains of the α-subunits form a kind of gate (pore) that opens or closes the access for the substrate to enter the catalytic cavity; α-subunits are also responsible for substrate recognition. Three of the seven β-subunits are catalytically active and provide proteolytic activity of the proteasome: β1-subunit has caspase-like activity (CL), β2-subunit has trypsin-like activity (TL), and β5-subunit demonstrates chymotrypsin-like activity (ChTL) [3,4]. Dysfunction of this complex can lead to regulatory proteins be stabilized due to reduced degradation or lost due to accelerated degradation [5]. Previous research has shown that tissue proteasome activity is associated with cancer progression and can be used to stratify risk [6,7].

Enzymatically active extracellular proteasomes have also been revealed in human plasma and cerebrospinal fluid [8,9,10]. Proteomic analysis of affinity-purified extracellular proteasomes revealed that extracellular proteasomes do not contain 19S or PA200 regulatory particles and are represented exclusively by the 20S complex [11]. Moreover, using matrix-assisted laser desorption/ionization Fourier transform ion cyclotron resonance mass spectrometry, was identified extracellular core 20S proteasome subunits and a set of 15 proteasome-interacting proteins, all previously described as exosome cargo proteins [11]. Taking this into account, as well as the fact that the 20S proteasome is a tetraspanin-unassociated protease of exosomes, it can be assumed that the most likely source of extracellular proteasomes are extracellular vesicles [11,12].

It is known that exosomes (endosome-derived vesicles (30–120 nm) [13]) are involved in such key physiological processes as intercellular communication [14], proliferation [15], migration [16,17], invasion [18], and angiogenesis [16,19] through horizontal transfer of RNA and proteins. On the one hand, exosomes are mini replicas of parent cells; on the other hand, selective transport of biologically active molecules has been shown in these vesicles [20]. It can be assumed that exosomes are one of the key players of tumor dissemination since they are very stable and small enough to penetrate from various tissues into biological fluids and then back, thus interacting with tissue target cells.

In our work we supposed critical role of exosomal 20S proteasome in mediating different types of metastasis. To test our hypothesis, we selected two female cancers (breast cancer and ovarian cancer) with different types of metastases (lymphogenic and peritoneal, correspondingly) and compared the enzymatic activity and the level of 20S proteasome in tissue and exosomes of healthy females and patients with ovarian and breast tumors.

## 2. Results

### 2.1. Characterization of Exosomes

The morphology of exosomes from blood plasma and ascites was examined by transmission electron microscopy. Presence of clearly structured cup-shaped particles of low electron density with a preserved membrane was revealed in exosome preparations isolated from blood plasma and ascites of breast cancer patients (BCPs), benign breast tumor patients (BBTPs), ovarian cancer patients (OCPs), benign ovarian tumor patients (BOTPs), and healthy females (HFs).

**Figure 1:**
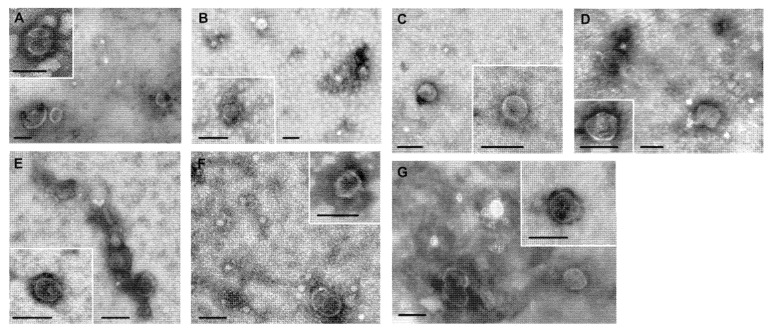
Total view of exosomes preparation obtained from: (**A**) blood plasma of HFs; (**B**) blood plasma of BBTPs; (**C**) blood plasma of BCPs; (**D**) blood plasma of patient with BOTPs; (**E**) ascites of patient with BOTPs; (**F**) blood plasma of OCPs; (**G**) ascites of OCPs. Scale bars correspond to 100 nm. Electron microscopy, negative staining by phosphotungstate acid.

Vesicles with damaged membranes did not exceed 12%, and the portion of microvesicles (with the size smaller than 30 nm), was no more than 16% (Figure 1).

**Figure 2:**
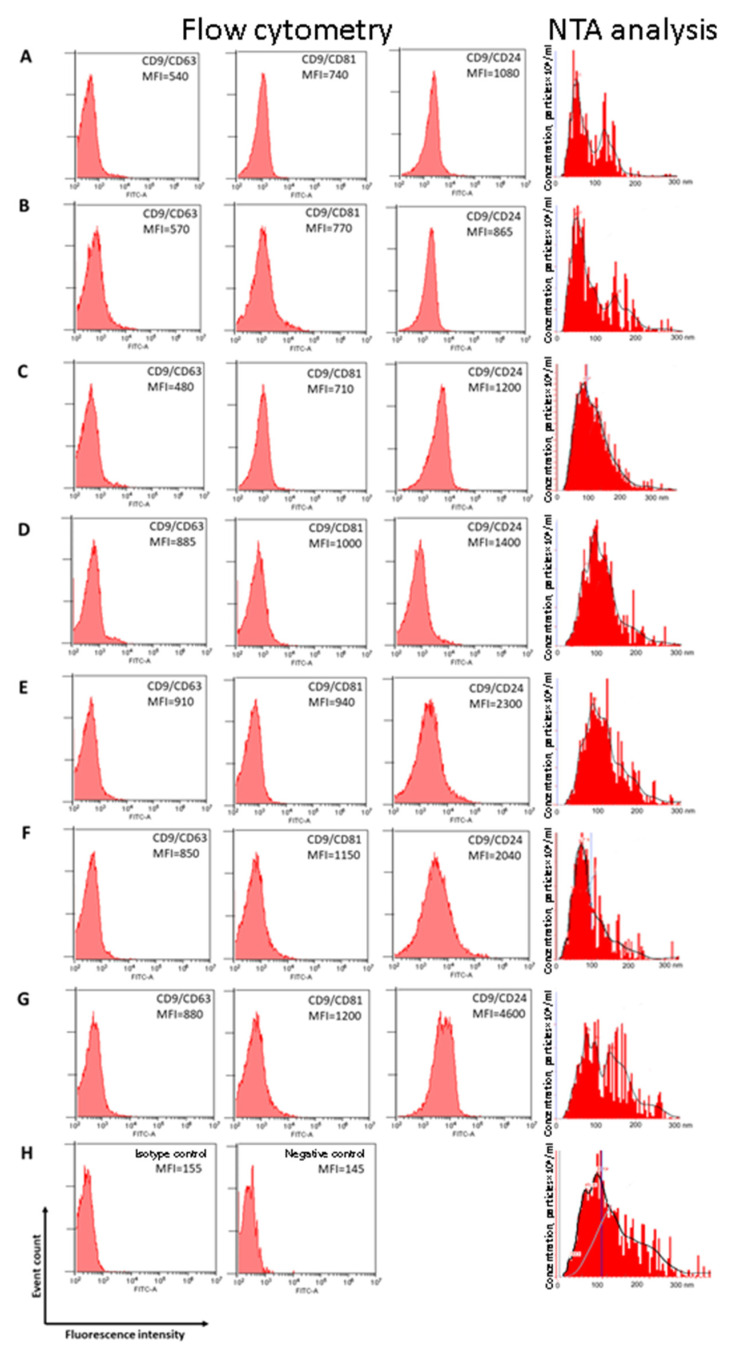
Exosomes characterization by flow cytometry and NTA analysis. For flow cytometry mean MFI are shown. Size distribution and expression of CD63, CD81, and CD24 on CD9-positive exosomes of (**A**) blood plasma HFs; (**B**) blood plasma of BBTPs; (**C**) blood plasma of BCPs; (**D**) blood plasma of OCPs; (**E**)ascitic fluid of OCPs; (**F**) blood plasma of patient with BOTPs; (**G**) ascitic fluid of patient with BOTPs; (**H**) Isotype control (left) and negative control (right).

Nanoparticle tracking analysis (NTA) showed that EVs from HF plasma had a mean size of 88 nm, with a mode of 55 nm and an SD of 45 nm (Figure 2A). Similarly, EVs from BBTP plasma had a mean size of 95 nm, with a mode of 53 nm and an SD of 59 nm (Figure 2B). EVs from cancer patients plasma were slightly larger than in normal plasma: mean size of 123 and 118 nm, with a mode of 87 and 96 nm and an SD of 64 and 53 nm for BCP plasma and OCP plasma, correspondingly (Figure 2C,D). The size distribution of EVs from ascites (a mean size of 135 nm, with a mode of 90 nm and an SD of 80 nm) and from plasma of patients with ovarian cancer are similar (Figure 2E); however, in ascites of BOTPs the size distribution of EVs (a mean size of 132 nm, with a mode of 76 nm and an SD of 67 nm) is similar to the superposition of EVs from BOTP plasma (a mean size of 110 nm, with a mode of 71 nm and an SD of 83 nm) and EVs from OCP ascites (Figure 2F,G). According to the most rigorous definition, exosomes are extracellular membrane particles of endocytic origin ranging from 30 to 130 nm emerging during the formation of multivesicular bodies and secreted into the extracellular space as a result of the fusion of multivesicular bodies with the plasma membrane. Microvesicles are a number of extracellular membrane particles that, in addition to exosomes, include apoptotic blebs—membrane particles with a diameter of 50 to 500 nm separated from the cells in the process of apoptosis, and shedding microvesicles—microparticles with a diameter of 100 to 1000 nm secreted by various cells under physiological conditions and probably playing an important role, along with exosomes, in the cell-to-cell communication and transport of proteins and nucleic acids [21]. To confirm the exosomal nature of isolated exosome-like vesicles, EVs were inspected for the presence of exosomal markers (CD9, CD24, CD63, and CD81) using flow cytometry. For this, vesicles absorbed into aldehyde sulfate latex beads using anti-CD9 antibodies were stained with FITC-labeled antibodies to tetraspanin family receptors CD63 and CD81, as well as to CD24 receptor. A subpopulation of CD9/CD24-positive exosome-like vesicles predominated in all samples (Figure 2).

Collectively, obtained data reveal that the extracellular vesicles isolated from blood plasma and ascites have all properties of exosomes [22].

### 2.2. Proteasomal Activity and the Level of 20S Proteasome in Tissues

In the study, the significant increase in chymotrypsin-like proteasomal activity (ChTL) and caspase-like (CL) proteasomal activity in malignant breast and ovarian tumors, was identified in comparison to the normal tissues (Table 1).

**Figure 3:**
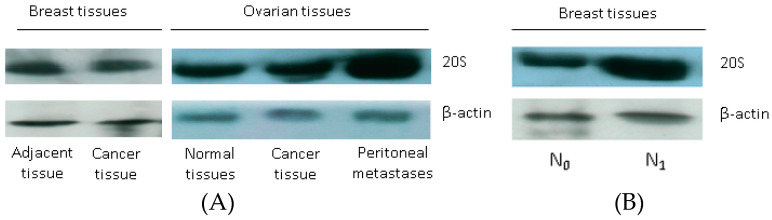
The 20S proteasome level in tissues, detected by Western blot. Western blotting was performed using antibodies against α&β subunits of 20S proteasome. β-Actin was used as the internal control. (**A**) Primary cancer tissues, adjacent breast tissue and normal ovarian and peritoneal metastasis tissues; (**B**) Luminal B subtype breast cancer tissue without (N_0_) and with regional lymph node metastasis (N_1_).

In particular, in breast cancer tissue the median values of ChTL and CL proteasomal activities were 2.3 and 3.0 times higher than in adjacent unchanged tissue. For ovarian cancer tissue the median values of these enzyme activities were 2.3 and 2.7 times higher than in normal ovary tissue too. It should be noted that the level of 20S proteasome was significantly increased in ovarian cancer tissue (at 1.7 times) and was comparable in breast cancer and adjacent unchanged breast tissue (Table 1, Figure 3A).

In total group of BCPs, there was no correlation of ChTL and CL activity of proteasome and the level of 20S proteasome in tumor tissue with age, molecular subtype, lymphovascular invasion and lymphogenous metastasis. Nevertheless, the level of 20S proteasome in tumor tissue was significantly higher (at 2.4 times) in patients with luminal B (HR + /HER2−) subtype with the presence of metastases in regional lymph nodes in comparison to patients without metastases (Table 2, Figure 3B).

At the same time, in patients with advanced ovarian cancer, the ChTL and CL activities of proteasome in the tissue of peritoneal metastases were significantly increased in comparison with the primary tumor (at 2.0 and 1.4 times, correspondingly) (Table 2, Figure 3A).

### 2.3. Proteasomal Activity and the Level 20S Proteasome in Exosomes from Plasma and Ascites of Tumor Patients

The ChTL proteasomal activity and CL proteasomal activity in exosomes from plasma of HFs and all patients, as well as from ascites of BOTPs and OCPs were lower than detection limit of assay (data not shown).

**Figure 4:**
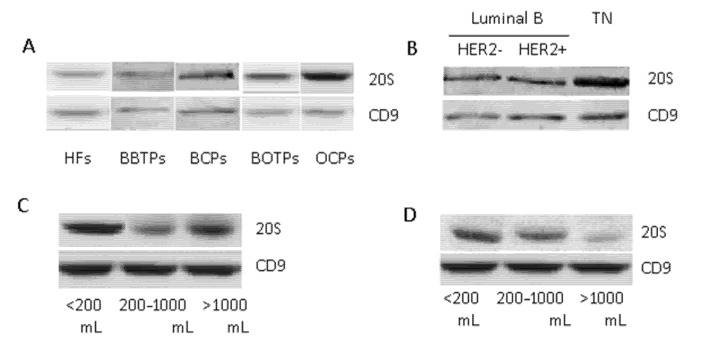
The 20S proteasome level in exosomes from plasma and ascites of tumor patients compared to healthy females, detected by Western blot. Western blotting was performed using antibodies against α&β subunits of 20S proteasome. CD9 was used as the internal control. (**A**) Plasma exosomes from HFs and patients with breast and ovarian tumors; (**B**) plasma exosomes from BCPs with Luminal B or TN molecular subtypes; (**C**) ascites exosomes from OCPs in depending on ascites volume; (**D**) plasma exosomes from OCPs in depending on its volume.

The 20S proteasome levels were significantly higher in BCP and OCP plasma exosomes (at 1.6 and 2.4 times, correspondingly) and did not differ in BBTP and BOTP plasma exosomes from plasma HF exosomes (Table 3, Figure 4A).

NTA analysis revealed an increased concentration of exosomes in the blood plasma of tumor patients in comparison with HFs. Moreover, the concentration of exosomes in the cancer patient plasma was lower than in patients with benign tumors (Table 3). However, these differences were not significant.

It was shown that the level of 20S proteasome in the blood exosomes of tumor patients is associated with molecular subtype of breast cancer. In particular, the level of exosomal 20S proteasome was significantly higher in the triple-negative subtype than in the less aggressive luminal B subtypes (HER2-positive and HER2-negative) (Table 4, Figure 4B). However, the relationship of 20S proteasome level in BCP plasma exosomes with age, lymphovascular invasion, and lymphogenous metastasis was not revealed.

The level of 20S proteasome was then investigated in plasma and in ascitic fluid in relation with ascites volume. The obtained data on the level of the exosomal proteasome from OCP plasma and from OCP ascites were compared with the corresponding samples from BOTPs. The level of ascites 20S proteasome was significantly higher in OCPs with low-volume ascites compared with the level observed in OCPs with moderate- and high-volume ascites (*p* = 0.048, Kruskal–Wallis test, Table 5, Figure 4C). A similar relationship was observed for blood plasma exosomes in OCPs, but the differences were not significant (*p* = 0.128, Kruskal–Wallis test, Table 5, Figure 4D). The relationship of 20S proteasome level in OCP plasma and ascites exosomes with age and stage of disease was not revealed.

Regression analysis was performed to assess the relationship between the proteasomal activity and level of the 20S proteasome in breast and ovarian cancer with the most important clinical and morphological parameters. Significant relationships were revealed between the 20S proteasome level in BCP plasma exosomes with molecular subtype of breast cancer (Table 6).

The lack of reliable relationships between the 20S proteasome level in OCP plasma exosomes and in OCP ascites exosomes with volume of ascites, can be explained by the insufficient size of patient groups (Table 5 and Table 6).

## 3. Discussion

Breast cancer and ovarian cancer occupy the leading places in the structure of cancer incidence in women, especially in industrialized countries [23]. In breast cancer, the definition of major molecular subtypes is often used in clinical practice to optimize the treatment of patients with both early and advanced cancer. Regardless of the molecular subtype of the tumor, the presence of lymphovascular invasion and lymphogenous metastases are prognostically unfavorable factors in breast cancer [24]. Ovarian cancer is characterized by high rates of one-year mortality, which indicates the aggressiveness of this tumor, which is mainly represented by G3 adenocarcinomas and is detected in 70% of cases at stage III [23]. Unlike lymphogenous metastatic neoplasms, tumor cells in ovarian cancer spread mainly inside the abdominal cavity. Malignant ascites constitute a tumor microenvironment that promotes migration and increased invasive activity of tumor cells due to the high content of cancerous exosomes and molecules with high biological activity (growth factors, cytokines, etc.). In OCPs, the presence of a large volume of ascites is associated with a low frequency of optimal cytoreduction and low overall survival [25].

Although malignant cells themselves are the major source of tumorigenesis, their interactions with the tumor microenvironment are critical for the progression from a single tumor mass to distant metastases [26]. It was shown that exosomes carry messages (proteins, non-coding RNA, DNA, lipids, metabolites) from transformed healthy cells or to other cells in the tumor or they may signal in an autocrine manner back to the producing tumor cells causing changes in the recipient cells behavior and microenvironment alterations [27,28,29,30,31]. Moreover, cargo of exosomes from tumor microenvironments such as ascites fluids has been found to be highly immunosuppressive [32,33,34], helping cancer progression and metastasis. Thus, it is probably the cargo of exosomes that is responsible for various types of metastasis (lymphogenous, peritoneal, etc.) of various malignant neoplasms.

We assessed the level of 20S proteasome and proteasomal activity both in tissues and in exosomes of healthy women and patients with female tumors to confirm for the first time the critical role of exosomal cargoes in mediating different types of metastases (lymphogenic and peritoneal). According to previous studies the significantly increased ChTL and CL proteasomal activities in ovarian cancer tissue were revealed in comparison to the normal tissues [7]. In addition, we revealed a significantly increased enzymatic proteasomal activity in the tissue of peritoneal metastases of OCPs. The level of 20S proteasome was significantly increased in cancer tissue and plasma exosomes from OCPs that reflect cell biology they stem from. These data are consistent with our previous study, which showed that 20S proteasome level was higher in samples of exosomes from SVO-3 culture medium than in exosomes from plasma OCPs and BOTPs as well as ascites of OCPs [35]. Moreover, it was found that the level of the 20S proteasome in plasma exosomes and ascites exosomes in patients with ovarian tumors is comparable, which can later be used to develop methods for evaluating the effectiveness of therapy and predicting the development of cancer. An unexpected artifact was the high level of 20S proteasome in the plasma exosomes and ascites exosomes from OCPs, which are characterized by a positive disease prognosis and a small volume of ascites. At the same time, these OCP exosomes have undetected enzymatic proteasomal activity, which may indirectly indicate the presence of proteasome inhibitors in their composition. Similar results were obtained for blood plasma exosomes from colorectal cancer patients: the level of 20S proteasomes in circulating exosomes was higher than in colorectal polyp patients, but the ChTL proteasomal activity was low or absent [36]. Moreover, in patients with Stage III colorectal cancer, the levels of 20S proteasomes (more than two units) and MMP9+ subpopulations (less than 60%) in plasma exosomes are favorable prognostic factors for overall survival. Another study found that despite comparable amounts of extracellular proteasomes from concentrated conditioned medium and cellular proteasomes from whole-cell extract of K562 cells, the ChTL, CL, and TL peptidase activities of affinity purified extracellular proteasomes were about 50%, 25% and 15% of cellular proteasomes, respectively. Authors suggest that proteasome activity in the conditioned medium is inhibited by an unknown protein reversibly bound to extracellular proteasomes [11]. It is known that proteasome inhibitors have immunosuppressive effects [37,38]. Taking this into account, as well as the absence of proteasome enzymatic activity in the exosomes of plasma and ascites of OCPs, revealed in the current work, it can be assumed that it is proteasome inhibitors that cause the immunosuppressive effect of tumor exosomes.

In total breast cancer tissue, a significant increase of ChTL and CL proteasome activities was revealed, although the level of 20S proteasome in the primary tumor did not differ from the level in the adjacent tissue; only luminal B HER2-negative subtype BCPs with lymphogenic metastasis were characterized by significantly increased 20S proteasome level in the tissue. Despite the high level of 20S proteasome in blood plasma exosomes of BCPs, as in the case of exosomes of OCPs, the level of proteasome enzymatic activity was lower than the detection limit of the assay. At the same time, the level of 20S proteasome in exosomes of patients with triple-negative subtype was significantly higher than in exosomes of patients with luminal B subtype of breast cancer, which is estrogen-positive. The obtained results are consistent with our earlier data on a higher level of the 20S proteasome level in samples of exosomes from plasma malignant and non-malignant breast tumors as compared to those of MCF-7 culture medium [35]. It is known that the ubiquitin–proteasome system is an important mechanism for the down-regulation of ER [39] and allows the rapid removal of ER to maintain a proper cellular balance of ER protein levels in response to hormonal stimulation. Our obtained results allow suggesting that the exosomal cargos, rather than the amount of exosomes, play causal roles in the development of cancer therapy tolerance [40].

We assume that the increased content of the 20S proteasome, which does not exhibit enzymatic activity both in the exosomes of breast cancer patients and in the exosomes of ovarian cancer patients, revealed in this study, aids in the delivery of intact biologically active proteins (transcription factors, enzymes, etc.) from transformed cells to targets cells. Regardless of the type of metastasis (lymphogenic or peritoneal) in recipient cells, exosome-delivered proteasome-inhibitor complexes can dissociate and the enzymatically active proteasome can be involved in the regulation of multiple cellular processes through the elimination of proteins and/or generation peptides involved in these processes [41].

## 4. Materials and Methods

### 4.1. Human Samples

Blood samples from HFs (*n* = 39, median age 40) were obtained from Novosibirsk Central Clinical Hospital. HFs did not have any female disorders (dysplasia, endometriosis, etc.) or any malignant diseases. Blood samples from BBTPs (*n* = 28, median age 42) and tissue and blood samples from BCPs (*n* = 80, median age 56) were obtained from Novosibirsk Regional Oncology Dispensary and Tomsk National Research Medical Center (Table 7).

Tissue, blood, and ascites samples from BOTPs (*n* = 20, median age 38) and from OCPs (*n* = 30, median age 56) were obtained from Tomsk National Research Medical Center (Table 8). Blood samples from all enrolled patients were collected before therapy. Tissue samples from BCPs were obtained as a result of the radical mastectomy or breast-conserving surgery, which was a first line of therapy. The volume of ovarian tumor patient’s ascites was measured clinically and by ultrasound examination. Three clinical subgroups of OCPs were formed: patients having low-volume (<200 mL, *n* = 8), moderate-volume (200–1000 mL, *n* = 8) and high-volume ascites (>1000 mL, *n* = 14). All BOTPs and OCPs underwent diagnostic laparoscopy for morphological verification and surgical staging as well as for sampling of tumor tissue and ascites.

Histological sections were prepared from paraffin blocks according to the standard method with the staining with hematoxylin-eosin. All ovarian cancers were characterized as high-grade serous adenocarcinomas.

### 4.2. Immunohistochemistry

Determination of the molecular subtype of breast cancer was carried out after assessing the receptor status in the operating material (expression of receptors for estrogen (ER) and progesterone (PR)), HER-2 status and the level of proliferative activity (expression of Ki67) in accordance with the St. Gallen Consensus Recommendation [42]. IHC was prepared as described [43]. For ER and PR expressions, the cases were classified as positive when nuclear immunoreactivity was in  ≥1% of tumor cells according to the American Society of Clinical Oncology/College of American Pathologists (ASCO/CAP) guidelines [44]. Sections stained with ER and PR were scored using the H-score method [45]. HER2 protein-positive status was defined as a score of 3  +  by IHC or 2  +  by IHC together with the confirmed c-erbB2 gene amplification by fluorescence in situ hybridization (FISH).

### 4.3. Clarified Tissue Homogenates

Tissue samples to determine the level of proteasome activity were collected at the stage of surgical intervention, frozen and stored at −80 °C. Breast and ovarian cancer tissues as well as samples of adjacent unchanged breast tissue of BCPs were used in the study. Tissue samples of a morphologically unchanged ovary were taken from healthy women operated for prophylactic purposes in the case of a BRCA mutation detection. Preparation of clarified homogenates was prepared as described [7].

### 4.4. Exosome Isolation

Venous blood (9 mL) was collected in K_3_EDTA spray-coated vacutainers (Improvacuter, Guangzhou, Guangdong, China, cat. no. 694091210), immediately mixed using a rotary mixer, placed at +4 °C and processed within an hour after blood taking. To isolate plasma exosomes by ultrafiltration and differential ultracentrifugation, the previously described protocol was used [16]. The pellet containing plasma exosomes was re-suspended in 150 μL of PBS.

Ascites fluid (18 mL) was collected in same vacutainers during surgery of BOTPs and OCPs, immediately mixed using a rotary mixer, placed at +4 °C and processed within an hour after ascites taking. Ascites fluid was centrifuged at 1200× *g* for 20 min at 4 °C, supernatant was transferred into a new tube, and centrifuged to pellet cell debris at 17,000× *g* at 4 °C for 20 min. Ascites supernatants were diluted in PBS (P4417, Sigma, Saint Louis, MO, USA) in a 1:3 ratio, passed through a 100 nm pore-size filter (Minisart high flow, 16553-K, Sartorius, Göttingen, Germany), and the filtrates were centrifuged for 90 min at 100,000× *g* (4 °C). Pellets were suspended in 12 mL PBS and again centrifuged for 90 min at 100,000× *g* (4 °C). Then supernatants were removed, and the pellets were resuspended in 300 μL PBS.

Exosomes samples from plasma and ascites fluid were frozen in liquid nitrogen and stored in aliquots at −80 °C until required. The aliquots were thawed once before use.

### 4.5. Exosomes Characterization

Morphology of the isolated extracellular vesicles was assessed by transmission electron microscopy (TEM) as described previously [17]. The initial volumes of plasma or blood for the study of EV using the TEM were 20 mL.

The size and concentration of EVs were determined by NTA using the NanoSight NS300 (Malvern, UK) as described previously [16].

To evaluate the protein concentration of exosomes, a NanoOrange Protein Quantitation kit (NanoOrange^®^ Protein Quantitation Kit, Molecular Probes, Eugene, OR, USA) was used in accordance with the manufacturer’s recommendations.

Quantitative analysis of the exosomal tetraspanines on the surface of the isolated extracellular vesicles was carried out using flow cytometry as described previously [46]. Flow cytometry was performed on the Cytoflex (Becman Coulter, BioBay, China), using CytExpert 2.0 Software. The MFI of stained exosomes was analyzed and compared to the isotype control (BD bioscience, Heidelberg, Germany).

### 4.6. Proteasome Activity Assay

ChTL and CL activities of the total pool of proteasomes (0.5–2 μL) were determined in clarified homogenates of tumor and untransformed tissues by the hydrolysis of a specific fluorogenic oligopeptide substrates Suc-LLVY-AMC [47] and Cbz-LLG-AMC, correspondingly [47,48]. Reaction mixture contained 20 mM Tris-HCl buffer (pH 7.5), 1 mM DTT, 5 mM MgCl_2_, 1 mM ATP and 30 μM specific fluorogenic oligopeptide substrate. The reaction was performed at 37 °C for 20 min and terminated with 1% SDS solution. A microplate reader with the excitation wavelength of 380 nm and the emission wavelength of 440 nm measured the fluorescence intensity. To evaluate the activity of admixture proteinases in samples, specific inhibitor of proteasomes, 5 μM MG132 (Sigma) was used in the same reaction mixture. The amount of enzyme that hydrolyzed 1 nmol specific fluorogenic oligopeptide substrate within 1 min at 37 °C was considered one activity unit. Specific activity of proteasomes was expressed in activity units per 1 mg protein. Protein content was determined according to Lowry’s method.

ChTL and CL activities of the total pool of proteasomes were performed in the same way. Specific activity of proteasomes was expressed in activity units per 1 μg exosomal protein.

### 4.7. Western Blot Analysis

Aliquots of exosomes (30 μL, 7 μg of exosomal protein) were incubated for 90 min on ice with 7 μL of lysis buffer (125 mM Tris- HCl (pH 7.5), 750 mM NaCl, 0.5% SDS, 5% Triton X-100), and 3 μL of protease inhibitors cocktail (1 mM Aprotinin (Roche, Mannheim, Germany), 0.1 mM Pepstatin A (Tocris Bioscience, Bristol, UK), 0.1 mM Leupeptin (Roche, Mannheim, Germany). Then, the samples were sonicated (Sonopuls mini20, MS1, ultrasonic sensor MS1, Bandelin, Berlin, Germany) during 1 min, then incubated with sample buffer at 95 °C for 7 min and centrifuged at 13,000× *g* for 5 min. After centrifugation samples were applied to a 13% PAA gel for SDS-PAGE electrophoresis according to Lemmli. After electrophoresis, proteins were transferred to the PVDF membrane (Immobylon, Millipore, Singapore). The membrane was blocked with 1X iBind Solution (Life Technologies, Carlsbad, Germany) and incubated with primary antibody to 20S proteasomes (Anti-Proteasome 20S α + β subunit antibody (ab22673, Abcam, London, UK), dilution 1:2000) and anti-CD9 antibody (ab134375, Abcam, London, UK, dilution 1:2000) at 4 °C, then the membrane was washed and incubated with secondary antibody (goat anti-rabbit IgG-HRP (Santa Cruz Biotechnology, Dallas, TX, USA), 1:5000 dilution) in accordance with the instruction with automated Western blotting device iBind Western Device (Life Technologies, Tel-Aviv, Israel) for 3 h. Further the membrane was incubated with the Amersham ECL Western blotting detection analysis system (GE Healthcare, Little Chalfont, UK). The visualization was performed on the system ChemiDoc Touch (Bio-Rad, Singapore). The density of the bands was estimated using “ImageLab” computer program. The obtained results were standardized with regard to the level of CD9 in exosomes and the level of 20S proteasome from HF blood exosomes or BOTP ascites exosomes.

Western blotting of 20S proteasome in tissue supernatants was performed in the same way, except for the procedures of the supernatant incubation with lysis buffer and sonication. The obtained results were standardized with regard to the level of actin in tissue homogenates and the level of 20S proteasome from unchanged breast and normal ovarian tissues.

### 4.8. Statistical Analysis

The data were processed by SPSS Statistica 10.0 software and presented as median and interquartile range (Me [Q25; Q75]). To evaluate the difference, either Mann–Whitney or Kruskal–Wallis test was used. Linear regression analysis was performed to confirm the relationship between enzymatic activity and proteasome expression in tissues and in exosomes with the most important clinical and morphological parameters. *p* values < 0.05 were considered statistically significant.

## Figures and Tables

**Table 1:** Proteasomal activity and the level of 20S proteasome in breast and ovarian cancer tissues.

Biological Sample	Enzymatic Activity, 10^3^ U/mg Protein		Level of the 20S Proteasome (α + β), RU
	ChTL	CL	
Breast tissues (all subtypes), T_1–2_N_0–1_M_0_			
Cancer tissues, *n* = 80	45.6 [19.8; 92.3] *	53.4 [18.4; 197.2] *	0.72 [0.3; 2.5]
Adjacent tissues, *n* = 80	19.8 [10.3; 42.1]	17.7 [8.3; 43.6]	1.0 [0.3; 3.1]
Ovarian tissues, T_3_N_x_M_0_			
Cancer tissues, *n* = 30	30.0 [26.0; 45.2] **	57.3 [13.6; 127.4] **	1.7 [0.8; 2.9] **
Normal tissues, *n* = 10	13.0 [7.8; 15.6]	21.3 [11.2; 51.4]	1.0 [0.5; 2.0]

Note: Median and range value [25; 75%], RU–related units. * Significant differences with adjacent breast tissue, *p* < 0.05; ** Significant differences with normal ovarian tissue, *p* < 0.05.

**Table 2:** Relationship of the proteasomal activity and level of 20S proteasome in luminal B-like (HR + /Her2−) breast cancer and ovarian cancer tissues with lymphogenic and peritoneal metastasis.

Biological Samples	Enzymatic Activity, 10^3^ U/mg Protein		Level of the 20S Proteasome (α + β), RU
	ChTL	CL	
Breast cancer tissue (Luminal B (HR + /HER2−) subtype)			
Cancer tissues, T_1-2_N_0_M_0_, *n* = 25	45.7 [28.7; 116.67]	124.2 [29.3; 256.2]	0.51 [0.3; 0.6]
Cancer tissues, T_1-2_N_1_M_0_, *n* = 24	42.4 [18.52; 111.9]	39.8 [23.8; 97.6]	1.2 [0.9; 2.6] *
Ovarian tissue			
Cancer tissues, *n* = 30	30.0 [26.0; 45.2]	57.3 [13.6; 127.39]	1.7 [0.5; 2.9]
Peritoneal metastases, *n* = 20	60.5 [49.6; 66.6] **	79.7 [44.6; 120.5] **	1.9 [0.90; 3.18]

Note: Median and range value [25; 75%], RU–related units. * Significant differences between breast cancer tissue without and with metastases, *p* < 0.05; ** Significant differences between primary ovarian tumor and peritoneal metastasis, *p* < 0.05.

**Table 3:** The level of exosomal 20S proteasome and concentration of plasma exosomes from healthy female and tumor patients.

Individuals	Level of the 20S Proteasome (α + β), RU	Median and Range of Exosomes Concentration × 10^7^/mL
HFs, *n* = 39	1.00 [0.90; 1.12]	16 [8; 20]
BBTPs, *n* = 26	1.05 [0.95; 1.16]	24 [20; 138]
BCPs, *n* = 80	1.59 [1.22; 1.61] *	21 [10; 180]
BOTPs, *n* = 18	1.07 [0.85; 1.22]	29 [17; 99]
OCPs, *n* = 30	2.42 [1.60; 4.58] *	22 [13; 154]

Note: Median and range value [25; 75%], RU–related units; * Significant differences between HFs and cancer patients, *p* < 0.05.

**Table 4:** The level of 20S proteasome in plasma exosomes from HFs and BCPs.

Individuals	Level of the 20S Proteasome (α + β), RU
HFs, *n* = 39	1.00 [0.90; 1.12]
Luminal B HER2-negative, *n* = 49	1.56 [1.22; 1.59] *
Luminal B HER2-positive, *n* = 12	1.59 [1.52; 1.73] *
TN, *n* = 19	1.94 [1.58; 2.67] *^,^**

Note: Median and range value [25; 75%], RU–related units; * Significant differences between HFs and BCPs, *p* < 0.05. ** Significant differences between Luminal B and TN breast cancer subtypes, *p* < 0.05.

**Table 5:** The level of 20S proteasome in exosomes from plasma and ascites of ovarian tumor patients.

Patients	Ascites Volume	Level of the 20S Proteasome (α + β), RU	
		Plasma	Ascites
OCPs, *n* = 30	<200 mL, *n* = 8	2.80 [2.38; 4.30] *	2.9 [2.39; 3.87] *
	200–1000 mL, *n* = 8	2.10 [1.07; 3.90]	1.10 [0.77; 2.50]
	>1000 mL, *n* = 14	2.00 [0.56; 3.36]	1.50 [0.88; 2.70]
BOTPs, *n* = 20		1.00 [0.87; 1.20]	1.00 [0.75; 1.44]

Note: Median and range value [25; 75%], RU–related units; * Significant differences between BOTSs and OCPs, *p* < 0.05.

**Table 6:** The proteasomal activity and the level of 20S proteasome in breast cancer and ovarian cancer: predicting tumor aggressiveness using regression analysis.

	B	Standard Error	*p*	95.0% Confidence Interval	
**Breast Cancer Tissues (All Subtypes)**					
Lymphovascular invasion					
ChTL	0.003	0.009	0.777	0.985	1.021
CL	0.010	0.010	0.319	0.991	1.029
20S proteasome level	−0.032	0.016	0.546	0.938	1.099
Multicentric type of tumor growth					
ChTL	−0.031	0.008	0.500	0.954	1.086
CL	−0.033	0.012	0.785	0.946	1.090
20S proteasome level	−0.033	0.013	0.913	0.943	1.093
Luminal B HER-2 negative subtype breast cancer tissues					
Lymphogenic metastasis					
ChTL	−0.005	0.244	0.983	0.616	1.606
CL	0.047	0.306	0.877	0.576	1.908
20S proteasome level	**0.011**	**0.015**	**0.049**	**0.932**	**0.999**
Plasma exosomes from BCPs					
Molecular subtype					
20S proteasome level	**0.266**	**0.117**	**0.038**	**0.017**	**0.515**
Lymphovascular invasion					
20S proteasome level	−0.664	0.341	0.071	0.264	1.004
Plasma exosomes from OCPs					
Ascites volume					
20S proteasome level	−0.006	0.017	0.058	0.133	0.656
Ascites exosomes from OCPs					
Ascites volume					
20S proteasome level	−0.066	0.135	0.056	0.764	0.995

**Table 7:** Clinical characteristics of BCPs.

	Luminal B		Triple Negative, *n* = 19	Total Patients, *n* = 80
	HER-2 Negative, *n* = 49	HER-2 Positive, *n* = 12		
Tumor stage				
T1	15 (31%)	4 (33%)	7 (37%)	26 (33%)
T2	34 (69%)	8 (64%)	12 (63%)	54 (67%)
Nodal status				
N0	25 (51%)	5(42%)	13(68%)	43 (54%)
N1	24 (49%)	7 (58%)	6 (32%)	37(46%)
Tumor Grade				
G2	49 (100%)	12 (100%)	19 (100%)	80 (100%)
Lymphovascular invasion				
Positive	10 (20%)	2 (17%)	4(21%)	16 (20%)
Negative	39 (80%)	10 (83%)	15(79%)	64(80%)
Multicentric type of tumor growth				
Positive	8(16%)	3 (25%)	0(0%)	11(14%)
Negative	41(84%)	9(75%)	19(100%)	69(86%)
BRCA status				
Negative	49 (100%)	12 (100%)	19 (100%)	80 (100%)

**Table 8:** General clinical characteristics of the BOTPs and the OCPs.

	BOTPs, *n* = 20	OCPs, *n* = 30
Histology		
Serous		30 (100%)
Other	20 (100%)	
FIGO (2013) staging		
I-II	16 (80%)	
IIIB	4 (20%)	5 (17%)
IIIC		25 (83%)
Tumor Grade		
High-grade		30(100%)
Not specify	20 (100%)	
BRCA status		
Negative		
Not specify	20 (100%)	30 (100%)
Ascites volume		
<200 mL		8 (26.5%)
200–1000 mL		8 (27.5%)
>1000 mL		14 (47%)
Not specify	20 (100%)	

## Data Availability

Not Applicable.

## Sample Availability

Samples of the compounds are not available from the authors.
